# The spatio-temporal patterns of the topsoil organic carbon density and its influencing factors based on different estimation models in the grassland of Qinghai-Tibet Plateau

**DOI:** 10.1371/journal.pone.0225952

**Published:** 2019-12-05

**Authors:** Shiliang Liu, Yongxiu Sun, Yuhong Dong, Haidi Zhao, Shikui Dong, Shuang Zhao, Robert Beazley

**Affiliations:** 1 School of Environment, Beijing Normal University, Beijing, China; 2 Research Institute of Forestry, Chinese Academy of Forestry, Key Laboratory of Tree Breeding and Cultivation of State Forestry Administration, Beijing, China; 3 Department of Natural Resources, College of Agriculture and Life Sciences, Cornell University, Ithaca, New York, United States of America; Sichuan Agricultural University, CHINA

## Abstract

The grassland soils of the Qinghai-Tibet Plateau (QTP) store a large amount of organic carbon because of the cold, humid climate, and topsoil organic carbon is quite sensitive to global climate changes. However, the spatio-temporal dynamics and factors that influence the soil organic carbon (SOC) on the QTP’s grassland are not understood well. Moreover, there are few comparative analyses of different approaches to estimate the QTP’ SOC. In this study, we estimated the storage and patterns of SOC density (SOCD) using several methods, including MODIS (moderate-resolution imaging spectroradiometer) retrieval, field data and previous empirical models (Models1-4, and soil organic matter (SOM)). And their relations with aboveground biomass, soil moisture, temperature, elevation, and soil conductivity were further explored. The results showed that SOC showed a similar variation trend in the different models, in which it decreased with increasing bulk density (BD) in the topsoil at 30 cm. For meadow and steppe grasslands, Models 1, 2, and 4 showed similar estimated values of SOCD, while Model3 had a lower value than them. SOC storage in the BD 3 and SOM methods had abnormal values, while the MODIS-NDVI, BD 1, 2, and 4 methods had similar SOC stock values for meadow and steppe grassland. Moreover, meadow grassland had a higher SOC storage than did steppe grassland, with means values of 397.9×10^10^ kg and 242.2×10^10^ kg, respectively. SOCD’s spatial distribution using MODIS-NDVI method differed clearly from the empirical models, with a significant tendency for spatial variation that increased from the northwestern to southeastern regions on the QTP. Therefore, based on the values estimated and spatial variation features, the MODIS-NDVI method may be a more feasible and valid model to estimate SOC. Moreover, the mean annual SOCD values during 2000–2015 showed an increasing trend, with a higher mean value in meadow and a lower mean value in steppe. Further, SOCD was correlated significantly and positively with aboveground biomass and soil moisture, and negatively correlated with elevation and soil conductivity. Increasing temperature had negative effects on SOCD, which was consistent with the global trend. These results indicated that topsoil moisture plays a key role in SOCD spatial patterns. Our results provide valuable support for the long-term estimation of SOCD in future research on the QTP.

## Introduction

Grasslands cover 32% of the earth’s vegetated area and account for 40% of the total area in China [1, 2]. They are important in global carbon cycle and carbon sequestration in both soil and vegetation [3]. With respect to carbon pool, grasslands store 44.09 Pg C in China, and alpine grasslands on the QTP hold approximately 54.5% of total grassland carbon [4]. Thus, grasslands play a key role in the global carbon cycle because of their high carbon storage and high potential feedbacks to climate warming [5–7]. Approximately 90% of the total carbon on the QTP’s grasslands is allocated to the soil, and constitutes approximately 50% of China’s SOC stock; thus, soil is regarded as the foremost carbon reservoir [8]. Moreover, as a particularly sensitive area with respect to possible effects of global climate change, particularly the rising atmospheric CO_2_ levels attributable to anthropogenic activities that have been occurring since the 1750s, increasing attention has been paid to the QTP grasslands’ SOC [3, 9]. Therefore, the QTP may be an ideal observation area to monitor SOC stock changes and assess environmental threats caused by human activities [10]. However, the spatio-temporal patterns of SOC on the QTP remain uncertain, as data from repeated inventories and sufficient field observations still are lacking.

Many scholars in China have carried out considerable research on SOC models of grasslands on the QTP, which generally have studied vertical distribution characteristics, spatial patterns, soil carbon storage, methods to measure the SOC pool, carbon sink, soil respiration, and so on [11–15]. However, the methods used to estimate SOC have a significant effect on the results’ accuracy and credibility of estimation result. After reviewing previous studies, we found that four methods largely have been used to estimate SOC in grassland, including literature reviews, field measurements, previous soil empirical modeling, and remote sensing of vegetation indices [14, 16]. Because of the difference of estimation methods, quality standards for sample collection and underground biomass estimation, the results of different methods may differ in certain ways. However, few studies have focused on comparative analyses of different methods to estimate the QTP’s SOCD.

Soil type and remote sensing methods are used widely to estimate SOC storage. First, the soil type method estimates the soil carbon pool by establishing regression equation models based on soil property datasets, such as bulk density (BD) and soil organic matter (SOM) [14, 17]. These models establish the relation between BD and SOC primarily, and are stable empirical models that many researchers have explored on the QTP [15, 18]. Moreover, models can describe SOC’s spatial distribution with better universality and predictability, and are applied widely in carbon stocks estimation [1, 18]. Nevertheless, these models’ shortcomings are that they are very complex involving many parameters, and it is difficult to obtain large-scale data, which results in higher evaluation values of SOCD [14, 19]. Thus, with the development of technology, increasing attention is being paid to remote sensing, which is widely used now to measure biomass and SOC stocks [16, 20]. Remote sensing method can overcome traditional estimations’ shortcomings and improve estimation accuracy greatly. Above all it can represent the spatial distribution and temporal dynamics of SOC effectively. Many studies have found that the Moderate Resolution Imaging Spectroradiometer (MODIS) NDVI products with high frequency are scientifically applicable [16, 20]. Therefore, remote sensing methods should be integrated with soil type methods to assess the long-term spatio-temporal variation of SOC on the QTP.

Environmental factors, such as climatic factors, topography, vegetation biomass and types, and soil texture, etc. are considered the important factors that influence SOC dynamics on the QTP’s grassland [18, 21–23]. Climatic factors, temperature and precipitation, control the balance between carbon input from plant productivity and carbon output by soil carbon decomposition [21]. Topography, including elevation, slope, aspect, or slope position, influence hydrothermal processes by controlling radiation, temperature and precipitation, and thereby affect the vegetation distribution and soil decomposition rate, which results in large variations in SOC stock [22, 24]. Vegetation type can affect SOC by controlling carbon inputs from plant residues, carbon decomposition, and carbon exchanges in the atmosphere [21]. Soil texture also affects SOC content and stabilization, because silt and clay content can provide physical protection that promote SOC’s aggregation [21, 23]. However, the relation between SOC and environmental characteristics has been investigated rarely in high-cold regions, and only a few scholars have tried to explore the relations between SOC stocks or density and temperature, soil moisture, or other soil properties on the QTP [9, 25–27].

Therefore, we conducted this study using a soil property dataset, MODIS-NDVI data during 2000–2015 and field survey data to examine: 1) SOCD and SOC’s storage features using different methods on the QTP; 2) SOCD’s spatial distribution on the QTP measured by different methods; 3) SOCD’s dynamics on the QTP during 2000–2015 using the MODIS-NDVI method; 4) the correlations among SOCD and soil temperature, moisture, and conductivity, aboveground biomass, and elevation using the MODIS-NDVI method.

## Materials and methods

### Study area

**Fig 1:**
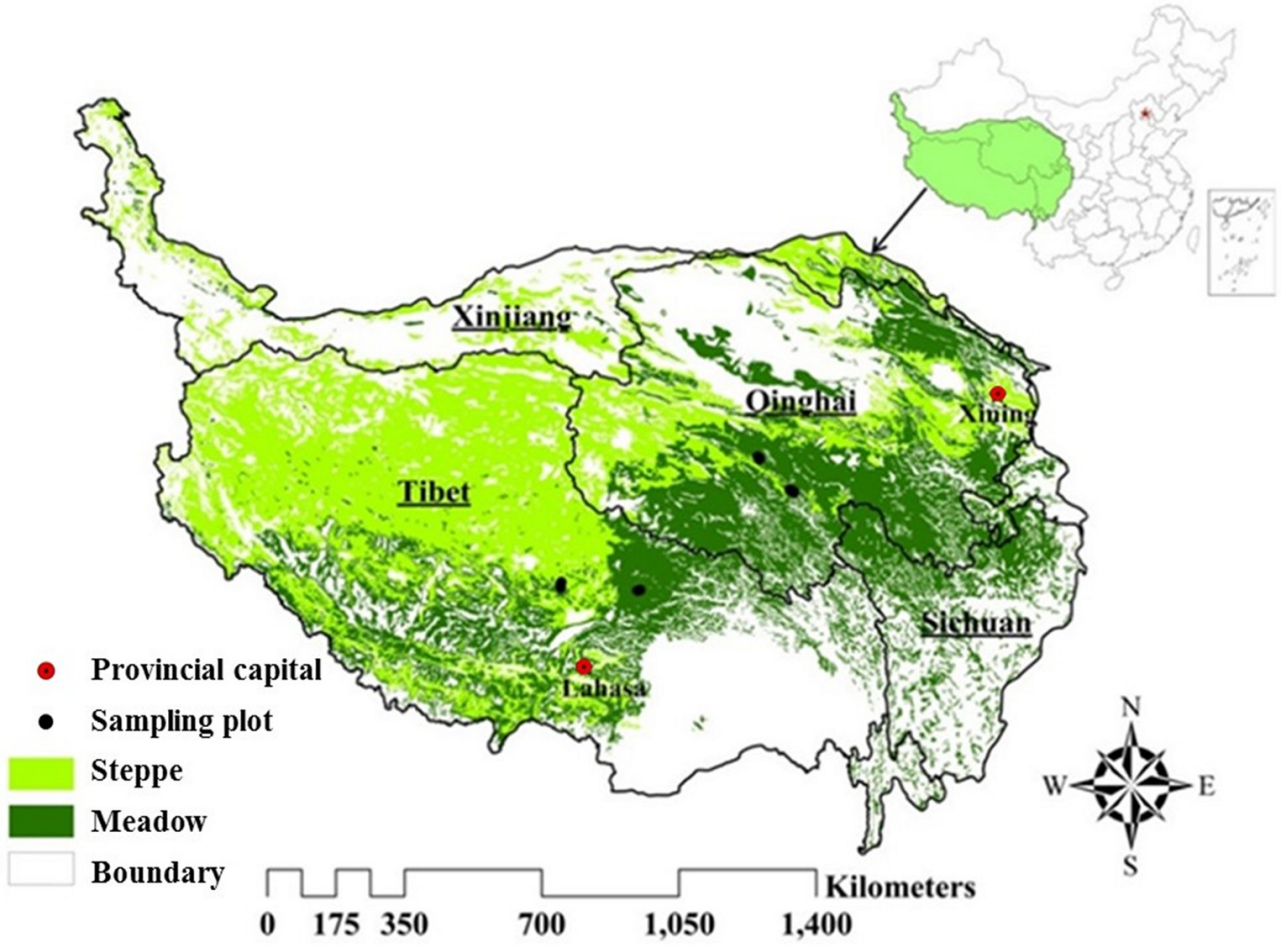
Location of the QTP and the distribution of steppe and meadow grassland.

This study was conducted on the Qinghai-Tibet Plateau (26°00’12”N–39°46’50”N, 73°18’52”E–104°46’59”E), the highest and largest plateau on earth with an average elevation of more than 4000 m. It is located in western China with an area of 2.61 × 106 km^2^, and encompasses the entire provinces of Tibet and Qinghai and part of the Xinjiang, Gansu, Sichuan and Yunnan provinces in China (Fig 1). Because of its complex terrain and variable boundary conditions, it is characterized by unique weather and climatic factors [28]. Its climate is warm and moist in the summer, and cold and arid in the winter, with a mean annual temperature of 1.61°C and annual precipitation of 413.6 mm [29]. The vegetation types in our study area include largely desert, steppe, meadow, tussock, and marsh, according to a vegetation map of 1:1000000 on the QTP. Alpine meadow and steppe grasslands are the dominant vegetation types and occupy for more than 60% of the QTP. The grassland types vary from meadow in the southeast to steppe in the northwest, where the climate varies from warm-wet in the southeast to cold-dry in the northwest (Fig 1).

### Data sources

#### Soil and vegetation sample collection

This field study is managed by Qinghai and Tibet forestry and grass bureau of China, state institutions. The institutions gave us permission to conduct this field study on the QTP, which did not involve any endangered or protected species. It was environmentally neutral and did not threaten the welfare of any species or that of the local population. Therefore, it was not related to ethical issues and no specific permissions were required for such activities.

A total number of 59 sample sites were visited during July and August 2013. 36 sample sites were recorded in Qumarleb and Nagchu counties, where the grassland type is meadow, and 23 sites in Quma river township and Baingoin county, where the grassland type is steppe. At each field sampling site, we selected a circle with 30m radius randomly and vegetation data were sampled using the methods Hankins et al. recommended [30]. In detail, three transects with nine 1m×1m quadrats were placed in each circle, and we obtained vegetation biomass data in each quadrat using the clip-harvest method [31], and also surveyed the vegetation types, height, frequencies, and coverage in each quadrat. The soil moisture, temperature, and conductivity data were obtained using the soil three-parameter speed detector (HW-WET-3), and the mean values measured at nine locations selected randomly were used to represent the soil condition in the sampling plots. The sampling sites’ locations were recorded using a handheld GPS (Fig 1) and certain environmental factors, including elevation, slope, aspect, etc. were also measured.

The soil characteristic dataset used was derived from the Second National Soil Survey with a 1:1 million soil map of China and 8595 soil profiles (available at http://westdc.westgis.ac.cn). The data primarily contains soil profile depth, soil thickness, BD, SOC (or SOM) and other physical and chemical properties [32]. The methods used to measure soil properties are described in the handbooks for soil surveys in China. Two principal methods were used to measure the organic matter fraction including the heating and the hydrothermal synthesis methods [32]. The two methods have the same principle, but the hydrothermal synthesis method use the heat from the chemical reaction rather than external heating [32]. Therefore, SOM was calculated with the following formula:

$$SOM=(a-b)N_{Fe}\times 0.003\times 1.724\times 1.08/W$$ (1)

where *SOM* is soil organic matter, *a* is the volume of a Fe^2+^ standard solution in the blank titration, *b* is the volume of a Fe^2+^ standard solution in the soil sample titration, *N*_*Fe*_ is the equivalent concentration of the Fe^2+^ standard solution, 0.003 is the carbon equivalent gram, 1.724 is the factor between SOC and SOM, 1.08 is the oxidation correcting coefficient, and *W* is the dry soil sample weight.

Because of the measurements’ limitation, BD was calculated from particle size distribution or estimated with the pedotransfer function. BD in the soil characteristic dataset was derived based on data measured from more than 1,000 profiles in China. Two primary methods were used to measure BD, including the ring (core) method and the wax immersion method [32]. And BD was calculated with the following equation:

BD=(M−G)×100/V/(100+W) (2)

where *BD* is bulk density, *M* is the cylinder and soil’s weight, *G* is the cylinder’s weight, *V* is the cylinder’s volume, and *W* is the percent of soil water content.

All data layers were expressed in raster format with a spatial resolution of 30 arc-second. To correspond the soil data with NDVI, soil samples were collected at depths of 0–9.1, 9.1–16.6, and 16.6–30 cm for each soil profile, soil layer1, 2 and 3, respectively.

#### NDVI data

**Fig 2:**
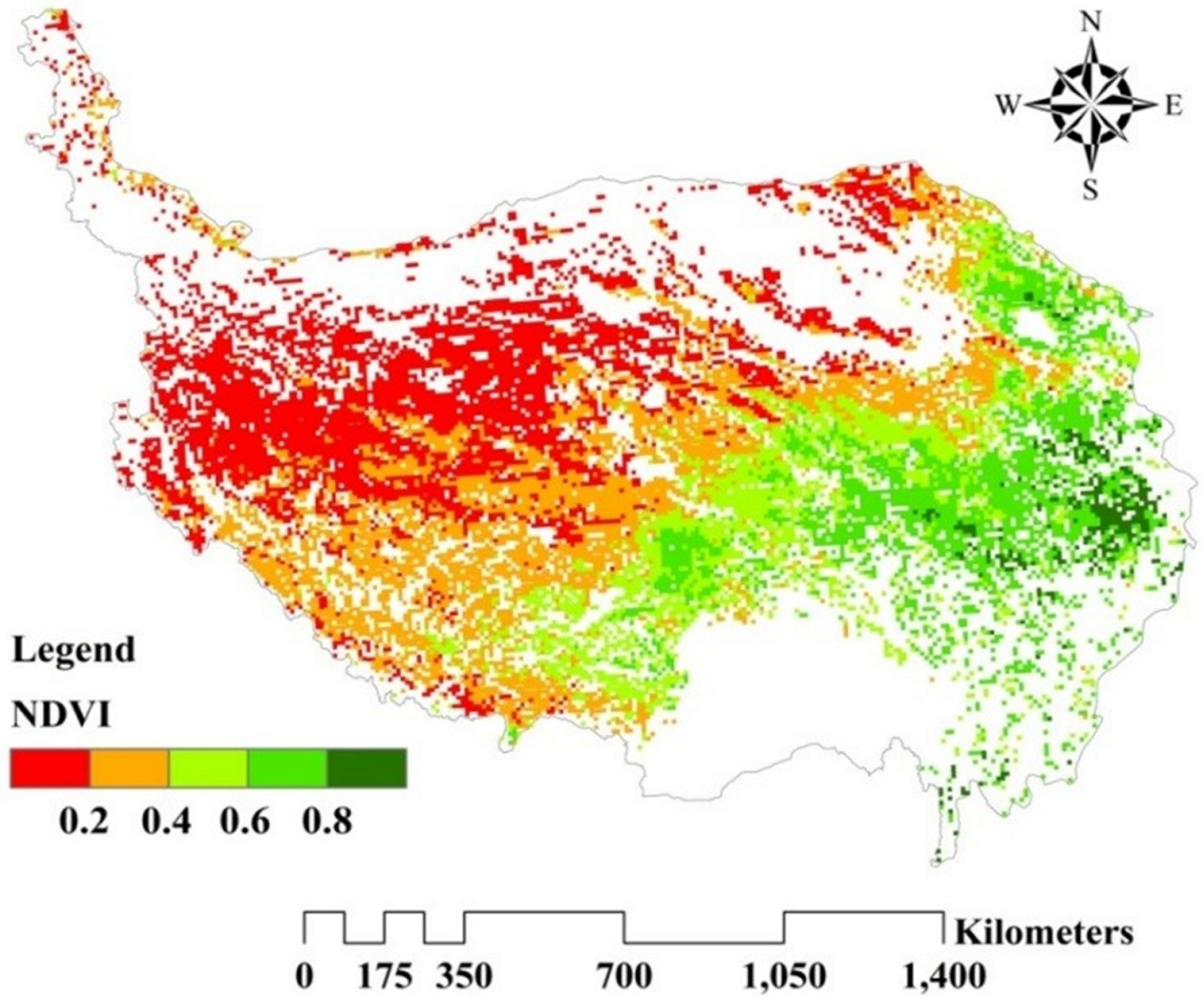
Spatial distribution of mean annual NDVI on the QTP during 2000–2015.

The MODIS-NDVI datasets used in the study were derived from the National Aeronautics and Space Administration (NASA) (available at http://daac.gsfc.nasa.gov/). The data from 2000 to 2015 had a spatial resolution of 1 km × 1 km and a 16-day temporal interval. The annual NDVI of each raster was obtained using the maximum value composite (MVC) method at the most abundant vegetation cover in a year. The adverse effects of cloud cover and certain errors attributable to large solar zenith angles could be diminished, which eliminated the seasonal variations’ adverse effects under different vegetation cover areas [33–35]. Then, using the aggregate method in the ArcGIS toolbox, the annual NDVI data from 2000 to 2015 with a 1 km × 1 km spatial resolution were replaced with 10 km × 10 km data. The steppe and meadow grassland’s mean annual NDVI distribution during 2000–2015 in the study area was mapped (Fig 2). The NDVI values showed a significant increasing trend in which the grassland type varied from steppe in the west to meadow in the east.

### SOCD calculation based on soil properties

#### SOCD calculation based on BD

The relation between BD and SOC is a relatively stable empirical relation that many researchers have explored on the QTP [14,31], and we calculated SOC by the inverse method based on BD to estimate SOCD for those soil profiles in the steppe and meadow grassland through four models published previously [14, 17, 36, 37], as follows:

Model 1 (BD 1):

$$BD=0.3+1.28\text{exp}(-0.01724SOC)\left({\text{R}}^{2}=0.66,p=0.0001\right)$$ (3)

Model 2 (BD 2):

$$BD=1.515\text{exp}(-0.102SOC)\left({\text{R}}^{2}=0.8654,\text{n}=65\right)$$ (4)

Model 3 (BD 3):

$$BD=0.9955+0.5427\text{exp}(-0.077SOC)({\text{R}}^{2}=0.46,\text{p}<0.001)$$ (5)

Model 4 (BD 4):

BD=1.4055×exp(-0.1039SOC) (6)

where *BD* and *SOC* represent bulk density (g· cm^-3^) and soil organic carbon (g· kg^-1^), respectively.

$$SOCD=\sum_{i=1}^{n}Ti\times BDi\times SOCi\times (1-C\text{i})/100$$ (7)

where *SOCD*, *T*_*i*_, *BD*_*i*_, *SOC*_*i*_, and *C*_*i*_ represent SOC density (kg·cm^-2^), layer thickness (cm), bulk density (g·cm^-3^), soil organic carbon (g·kg^-1^), and percentage of the fraction>2mm, respectively.

#### SOCD calculation based on SOM

The relation between SOM and SOC was transformed by a coefficient, in which the conversion factor depends on SOM’s carbon content rate. The “van Bemmelen factor” of 0.58 is still used in China, and we used its empirical equations by an inverse method based on SOM to estimate SOC and SOCD in the steppe and meadow grassland [14].

SOM=SOC×0.58 (8)

$$SOCD=\sum_{i=1}^{n}Ti\times BDi\times SOCi\times (1-C\text{i})/100$$ (9)

where *SOCD*, *SOM*, *T*_*i*_, *BD*_*i*_, *SOC*_i_, and *C*_*i*_ represent SOC density (kg·cm^-2^), soil organic matter (g·kg^-1^), layer thickness (cm), bulk density(g·cm^-3^), soil organic carbon (g·kg^-1^), and percentage of the fraction>2mm, respectively.

#### SOCD change trend analysis

Linear regression analysis is widely used to estimate vegetation change [38, 39], and we applied it to evaluate SOCD change in our study. The slope of the linear regression in the analysis was the index that quantified the SOCD change trend in the study period [40]. The slope was calculated as follows:

$$Slope=\frac{n\times \sum_{i=1}^{n}i\times SOCD_{i}-\sum_{i=1}^{n}i\sum_{i=1}^{n}SOCD_{i}}{n\times \sum_{i=1}^{n}i^{2}-{\left(\sum_{i=1}^{n}i\right)}^{2}}$$ (10)

where *n* is the number of study years (13 in this study), *i* is the serial number of the year, and SOCD_i_ is the SOCD value of year *i* in the grid. A positive slope in the grid corresponds to a positive trend in SOCD change over the 13 years, and a negative value corresponds to a decreasing trend.

The nonparametric Mann-Kendall (M-K) test is applied to determine the SOCD change trends and to quantify their statistical significance for each pixel. We estimated the values of Kendall inclination β and the Z statistics. β is an unbiased estimate for long-term trends, when β > 0, the sequence shows a significant upward trend; when β = 0, the sequence shows no significant trend; and when β < 0, the sequence shows a significant downward trend [41]. Based on the Z values from MK test, when Z > 1.96, the results have significant increasing; when Z < −1.96, the results have significant decreasing; and when −1.96≤Z≤1.96, the results have non-significant changes.

### SOCD calculation based on the NDVI method

Yang et al [17, 27] explored the relation between NDVI and SOCD on the QTP, and we used their established equations to evaluate SOCD in the steppe and meadow. The validity was evaluated by the correlation coefficients between grassland SOCD and aboveground biomass.

$$SOCD_{steppe}=20.201NDVI-0.9206$$ (11)

$$SOCD_{meadow}=17.846NDVI+0.0155$$ (12)

where *SOCD*_*steppe*_ and *SOCD*_*meadow*_ represent soil carbon density (kg·C·m^-2^) in the top 30cm of soil in the steppe and meadow, respectively.

### Correlation between SOCD and influencing factors

The SOCD values of the sampling plots were calculated based on the NDVI values in the same place and time. The correlation coefficients between grassland SOCD and soil moisture, temperature, and conductivity, aboveground biomass, and elevation were calculated using SPSS and the correlations between different factors were also estimated with the following formula:

$$r=\frac{\sum_{i=1}^{n}\left(x_{i}-\bar{x}\right)\left(y_{i}-\bar{y}\right)}{\sqrt{\sum_{i=1}^{n}{\left(x_{i}-\bar{x}\right)}^{2}\sum_{i=1}^{n}{\left(y_{i}-\bar{y}\right)}^{2}}}$$ (13)

where *n* is the number of sample plots, *y*_*i*_ is the value of the influencing factors in sample *i*, such as soil moisture, temperature, and conductivity; $\bar{y}$ is the mean values of these factors in all sample plots; *x*_*i*_ is the SOCD in sample plot *i*; $\bar{x}$ is the mean value of SOCD in all of the sample plots. When the values of *r* are significant (*p* < 0.01 or *p*< 0.05), it indicates that there is an “extremely significant” or “significant” linear correlation between SOCD and the analytic factor [42].

**Fig 3:**
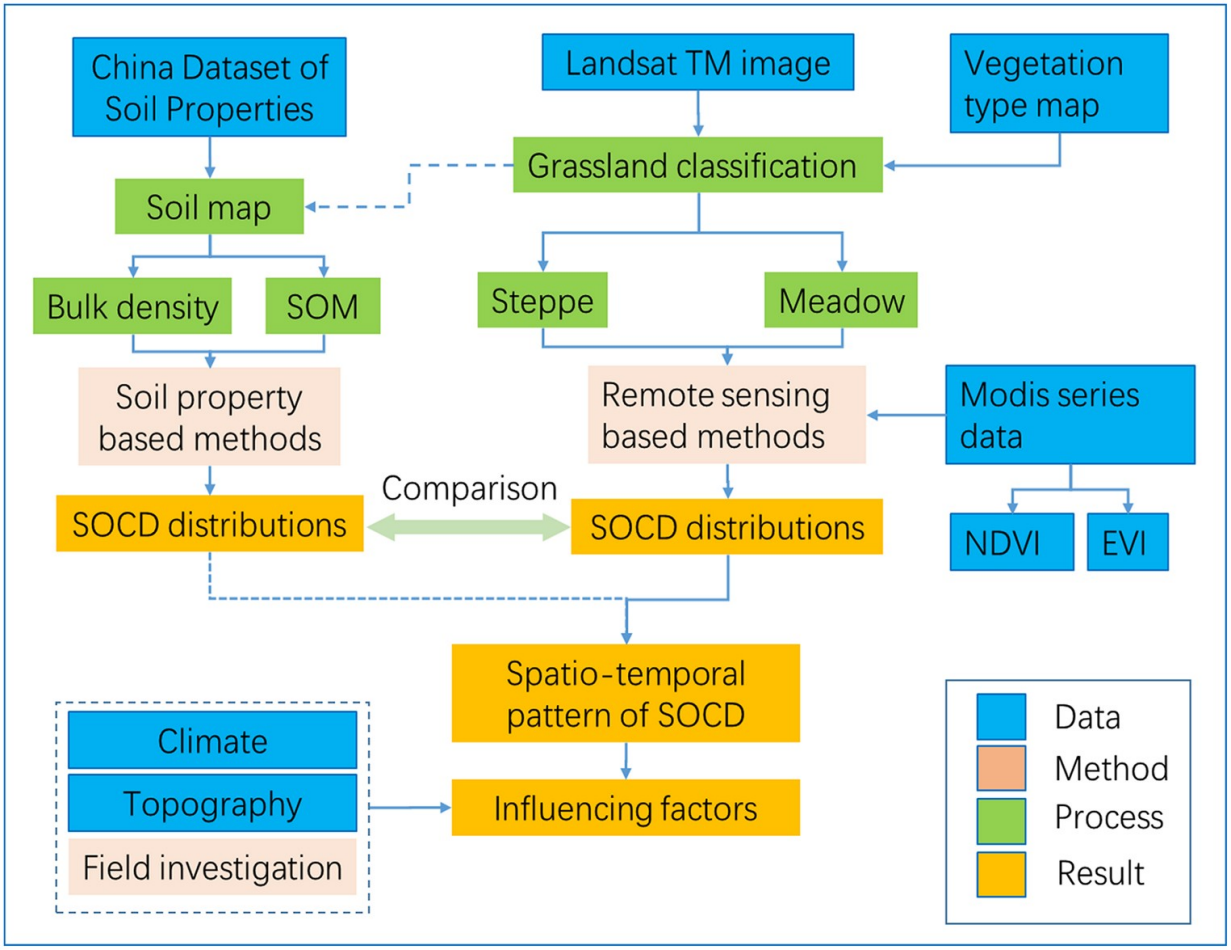
The technical route in this study.

The technical route of the study is shown in Fig 3.

## Results

### SOC density features on the QTP grasslands

**Fig 4:**
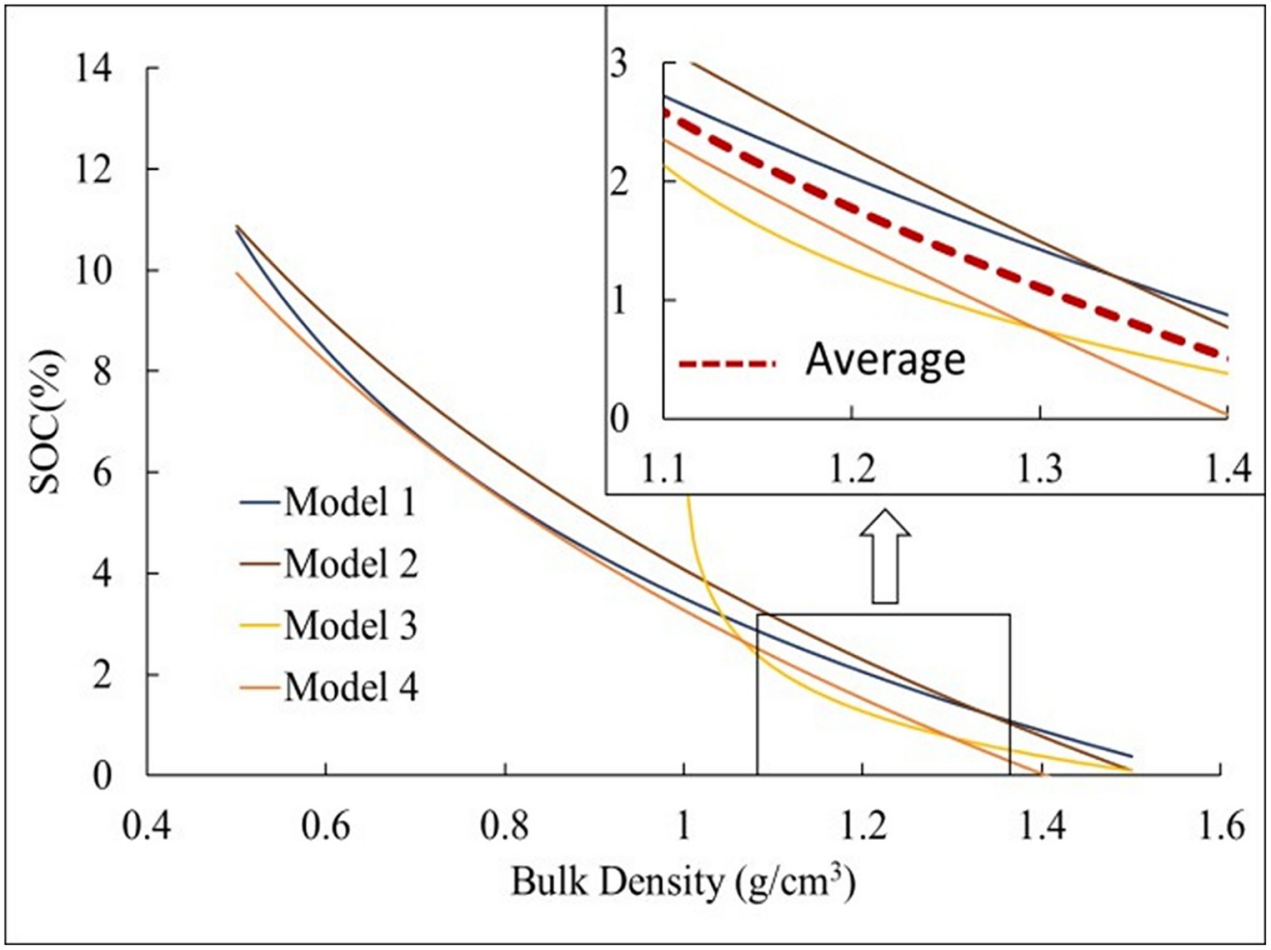
Model-simulated SOC based on bulk density for different models (Models1-4).

Fig 4 showed differences among the curves between the different models, but the variation between SOC and bulk density was similar, in which, in general, SOC decreased with increasing BD overalll. In this study, Model 3 differed clearly and unreasonably from other three models when BD was less than 1.1 g·cm^-3^, while it had a similar decreasing tendency with the other three models when BD was more than 1.1 g·cm^-3^. This may be attributable to the variations in the empirical formula based on different soil profile depths and sampling sites [36]. On average, from 1.1 to 1.4 g·cm^-3^ of BD, the four models exhibited a close and cross trend with a range of 1% difference. The SOC in Models 3 and 4 was below average, and that in Models 1 and 2 were above average.

**Fig 5:**
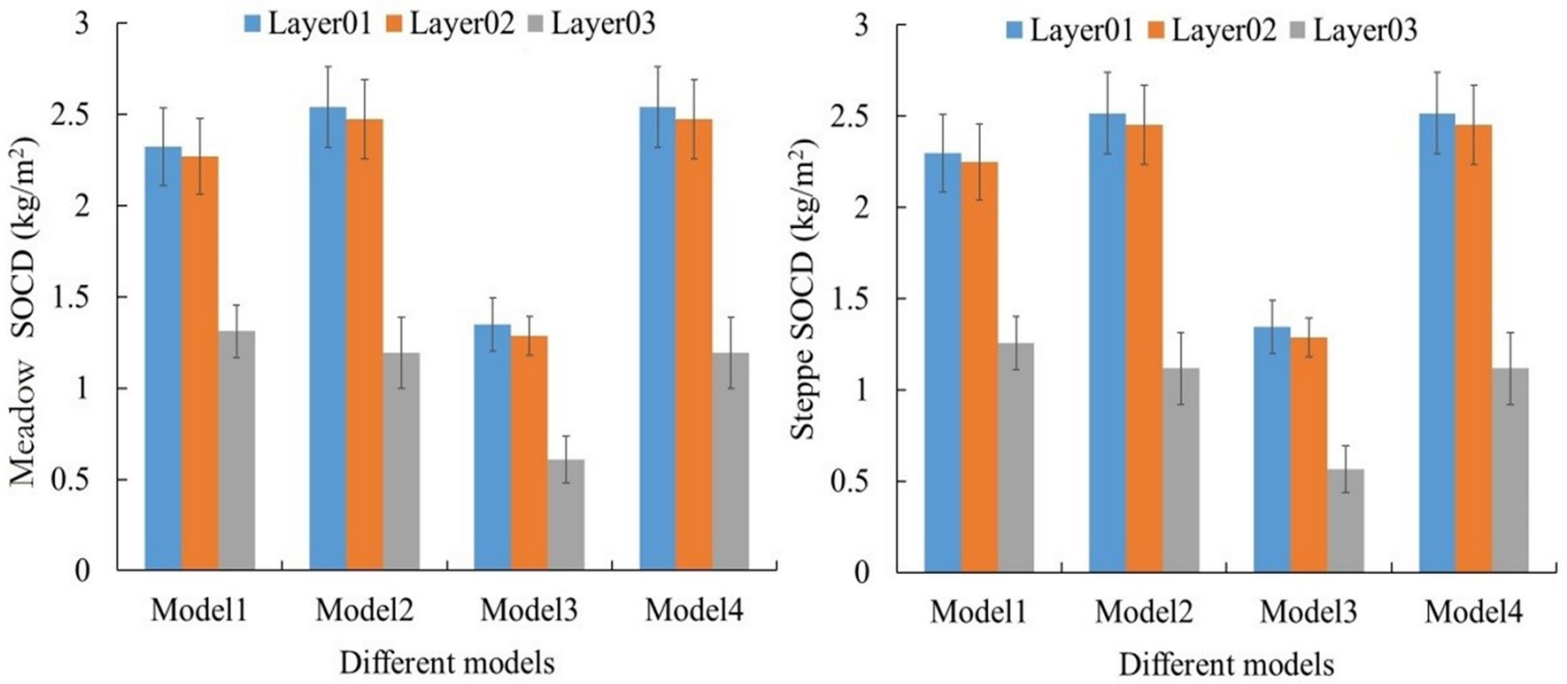
Different models of SOCD for steppe and meadow grassland with different soil layers (Layers 1, 2 and 3) on the QTP.

The SOCD on the QTP’s meadow and steppe grasslands was further calculated using the BD and SOC data in all soil layers (Fig 5). The Fig 5 showed that there were no significant differences among Models 1, 2 and 4 for meadow and steppe grassland, with similar SOCD values of 5.9 kg·cm^-2^, 6.1 kg·cm^-2^, and 6.1kg·cm^-2^ in total soil layers, respectively. However, compared with the three other models, Model3 differed clearly and had the lowest value, 3.2 kg·cm^-2^. Thus, Model3 may not be a suitable and valid estimation model, as it showed a large decrease on the QTP’ grassland. Further, for each soil layer in the different models, layers1 and 2 did not differ markedly, with a mean SOCD value of 2.2 and 2.1 kg·cm^-2^, while layer 3 was clearly lower with a value of 1.0 kg·cm^-2^. Finally, SOCD showed no significant differences between different grassland types, with mean values of 5.39 kg·cm^-2^ in the meadow grassland and 5.29 kg·cm^-2^ in the steppe grassland, respectively.

**Fig 6:**
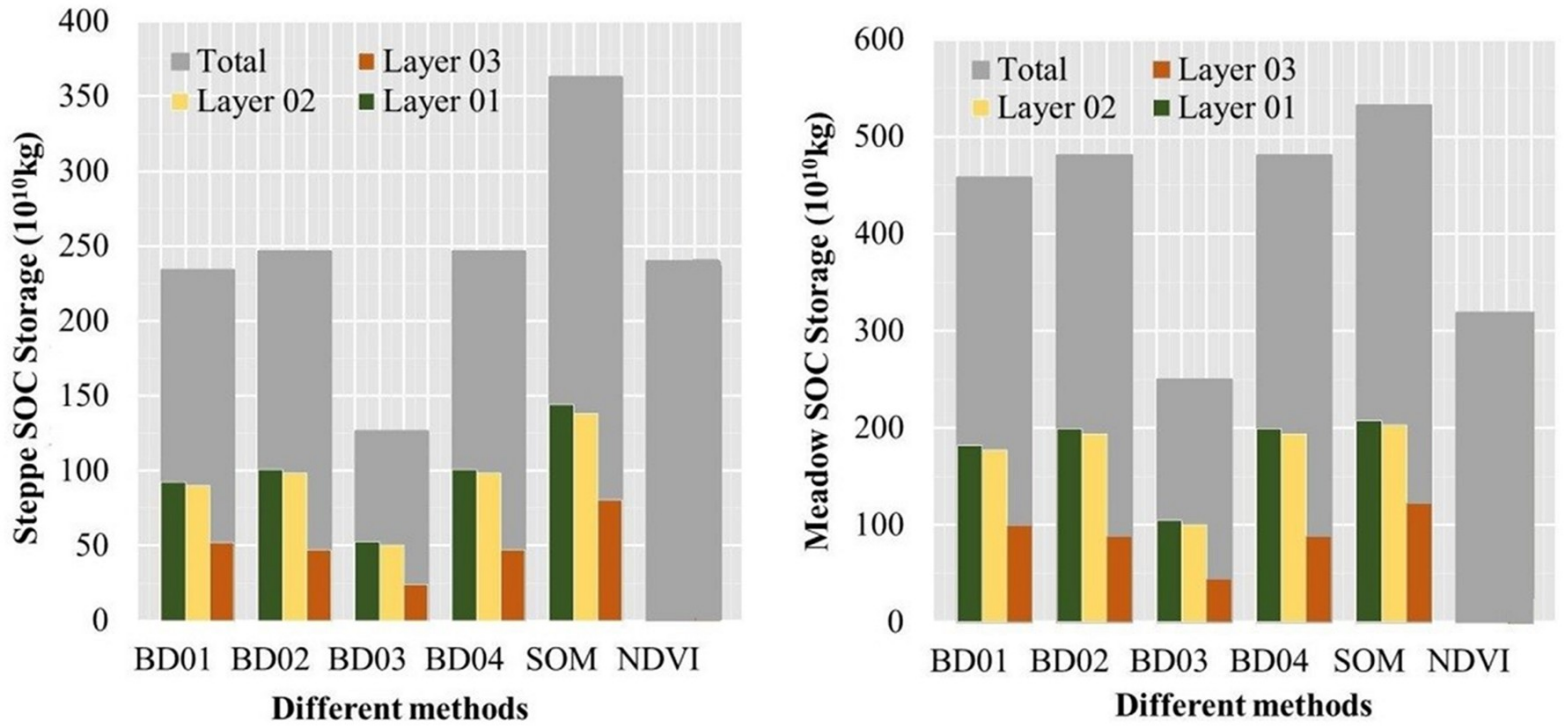
Different methods of measuring steppe and meadow grassland’s SOC storage at different soil layers (Layers1, 2, 3, and Total) on the QTP.

Fig 6 showed the total SOC stocks on the QTP for both meadow and steppe grasslands using the BD, SOM, and MODIS-NDVI methods, respectively. In general, for the total SOC stocks, the mean SOC storage in the meadow and steppe grasslands was the highest with the SOM method (448×10^10^ kg) and lowest with BD 3 method (188.3×10^10^ kg) (Fig 6). Moreover, for the steppe grassland, the SOCD result using the MODIS-NDVI method (240.2×10^10^ kg) was very similar to those with the BD1, 2 and 4 methods (234.4×10^10^ kg, 246.4×10^10^ kg, and 246.4×10^10^ kg, respectively), while for the meadow grassland, the SOCD result using the MODIS-NDVI method (319×10^10^ kg) was significantly lower than those using the three BD methods above (458.4×10^10^ kg, 481.0×10^10^ kg, 481.0×10^10^ kg, respectively) (Fig 6).

Further, Fig 6 also showed that SOC storage varied greatly among the different grassland types, and on the whole, SOC storage was higher in the meadow than in the steppe grassland (397.9×10^10^ kg and 242.2×10^10^ kg in total, respectively). Similarly, for different methods, SOC storage in the meadow grassland was obviously higher than that in the steppe grassland, and total SOC stocks were 458.4 ×10^10^ and 234.4×10^10^ kg, 481.0×10^10^ and 246.4×10^10^ kg, 250×10^10^ and 126.7×10^10^ kg, 481.0×10^10^ and 246.4×10^10^ kg, 532.6×10^10^ and 363.3×10^10^ kg, 319.0×10^10^ and 240.2×10^10^ kg, respectively. Finally, for each soil layer measured with the different methods, there were no significant differences in the SOC storage values between soil layers 1 and 2 (138.2×10^10^, 134.4×10^10^ kg, respectively), but they were clearly higher than the soil layer 3 (69.5×10^10^ kg), which corresponded to a decrease of 49.71%. Therefore, mean SOC storage also decreased with increasing soil depth in the topsoil at 30 cm.

### Spatial distribution of SOCD on the QTP’s grasslands

**Fig 7:**
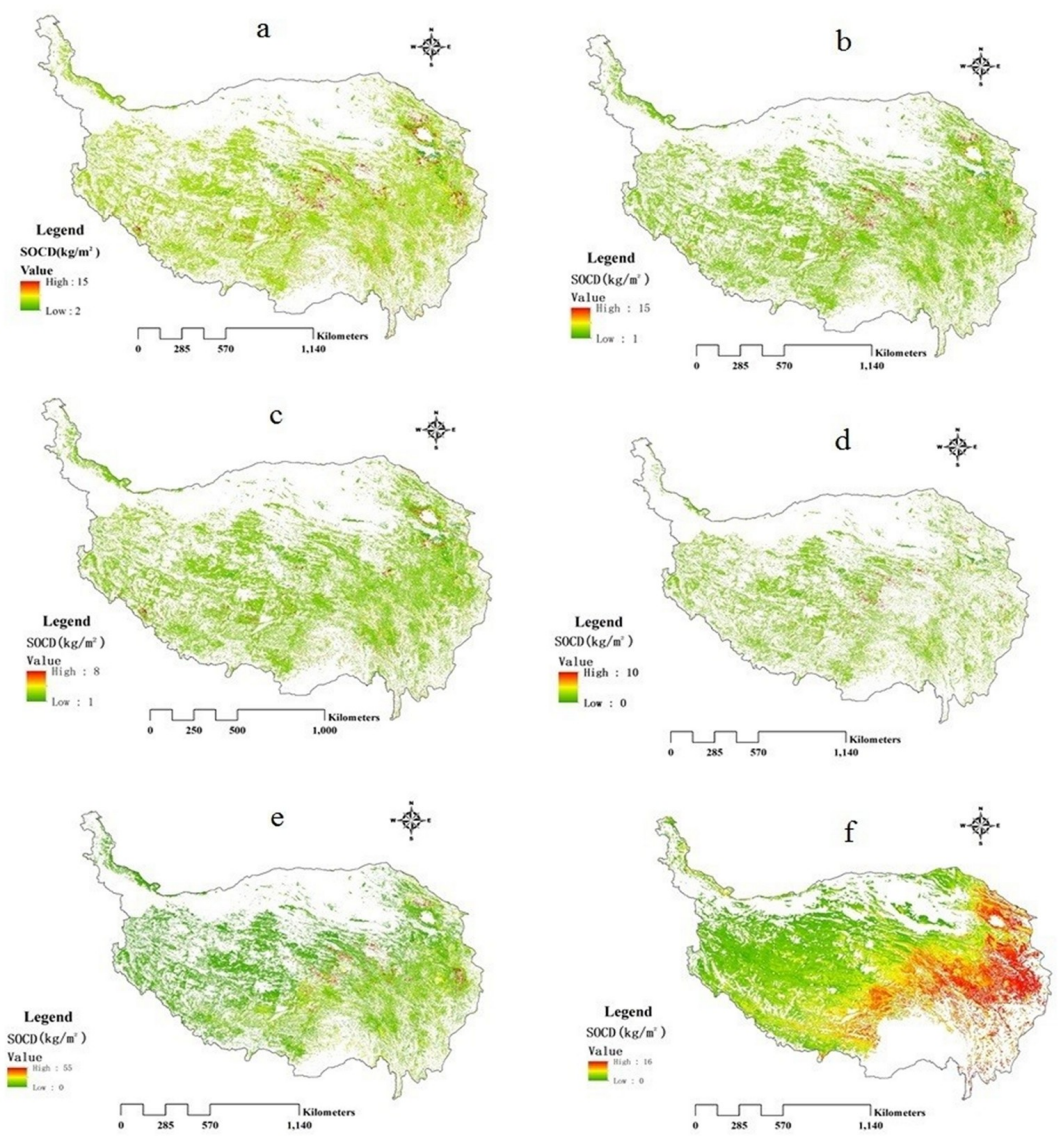
Spatial distribution of mean annual SOCD on the QTP with different methods. (a-d): Models1-4 methods; (e): SOM method; (f): MODIS-NDVI method.

We estimated the mean spatial patterns in the SOCD on the QTP’s meadow and steppe grasslands using Models1-4, SOM and the MODIS-NDVI methods (Fig 7). SOCD’s mean spatial distribution for Models1-4, and SOM showed a similar variation gradient, with higher values largely in sporadic areas in the mid-east (Fig a-d and e). From the middle to west or east, SOCD all showed irregular variations. However, SOCD’s mean spatial distribution between MODIS-NDVI and the five other models largely was heterogeneous. Fig 7f suggested that using the MODIS-NDVI method reflected SOCD’s spatial distribution and variation effectively, while the other five models showed no obvious and regular spatial variation trends. Therefore, in view of the spatial distribution and value range, the MODIS-NDVI method may be a more appropriate method to estimate SOCD, although there were still some shortcomings.

Further, SOCD showed a similar pattern in gradient variation as the NDVI values, in which there was an obvious increasing trend from northwest to southeast (Fig 7). SOCD was generally lower (below 4 kg·C·m^-2^) in northern Tibet, most of the Xinjiang province and northwest of the Qinghai province, which occupied for 44.31% of the total study area, and contained a greater proportion of steppe grasslands. However, SOCD was higher in the western areas of Sichuan and the southeastern Qinghai province, which accounted only for 12.20%, and contained a greater proportion of meadow grasslands.

### SOCD change trend on the QTP’s grasslands

**Fig 8:**
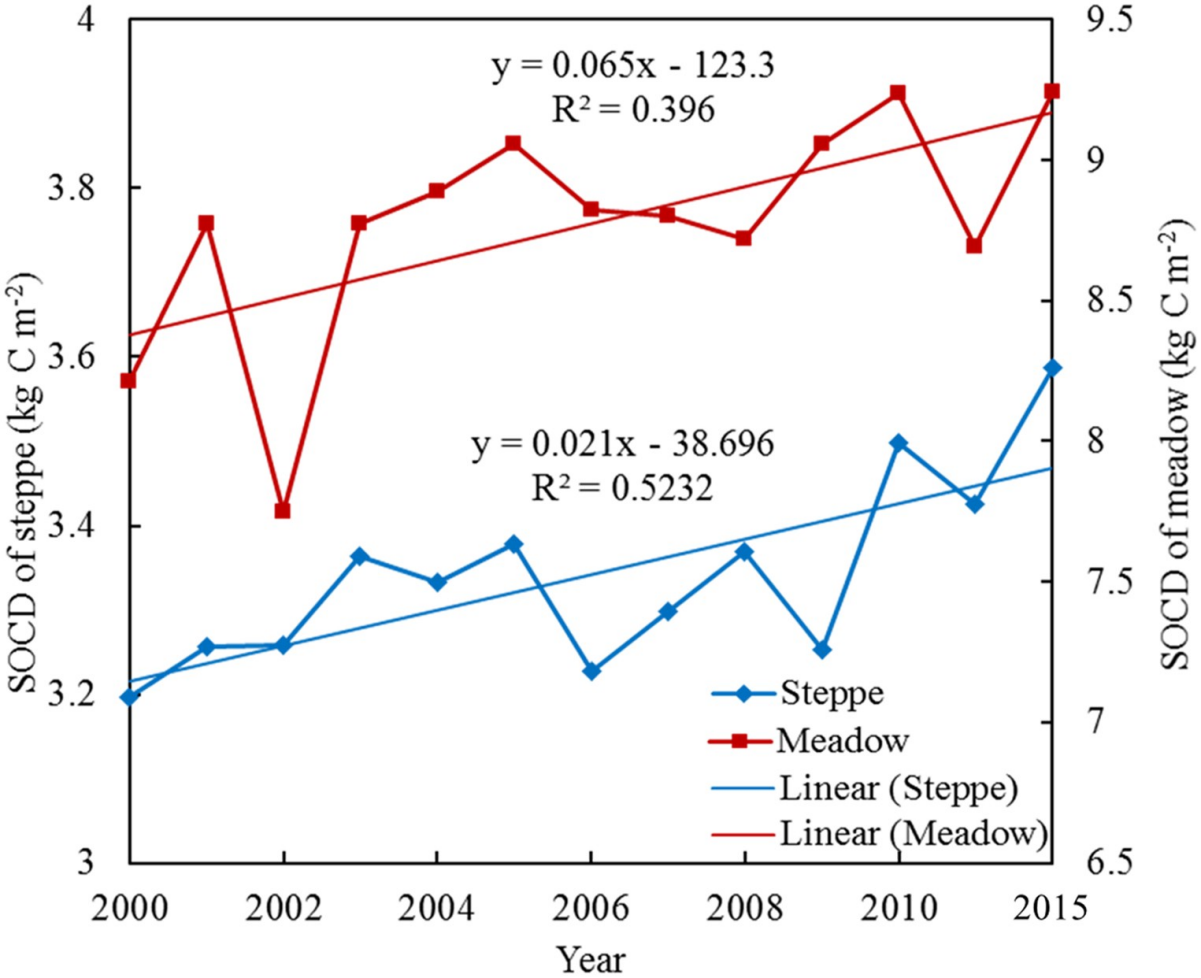
Annual average SOCD of steppe and meadow on the QTP during 2000–2015.

Temporally, the NDVI method revealed the SOCD dynamics more explicitly across the QTP. Generally, the annual SOCD of either the steppe or the meadow grassland increased during the study period (Fig 8). There was a significant upward trend in the annual average SOCD of the grassland on the QTP during 2000–2015, with a rate of 0.021 in the steppe and 0.065 in the meadow, respectively. The lowest SOCD value in the steppe was 3.20 kg·C·m^-2^ in 2000, while it was highest in 2015. However, the annual average SOCD of the meadow was lowest in 2002 with a value of 7.75 kg·C·m^-2^.

**Fig 9:**
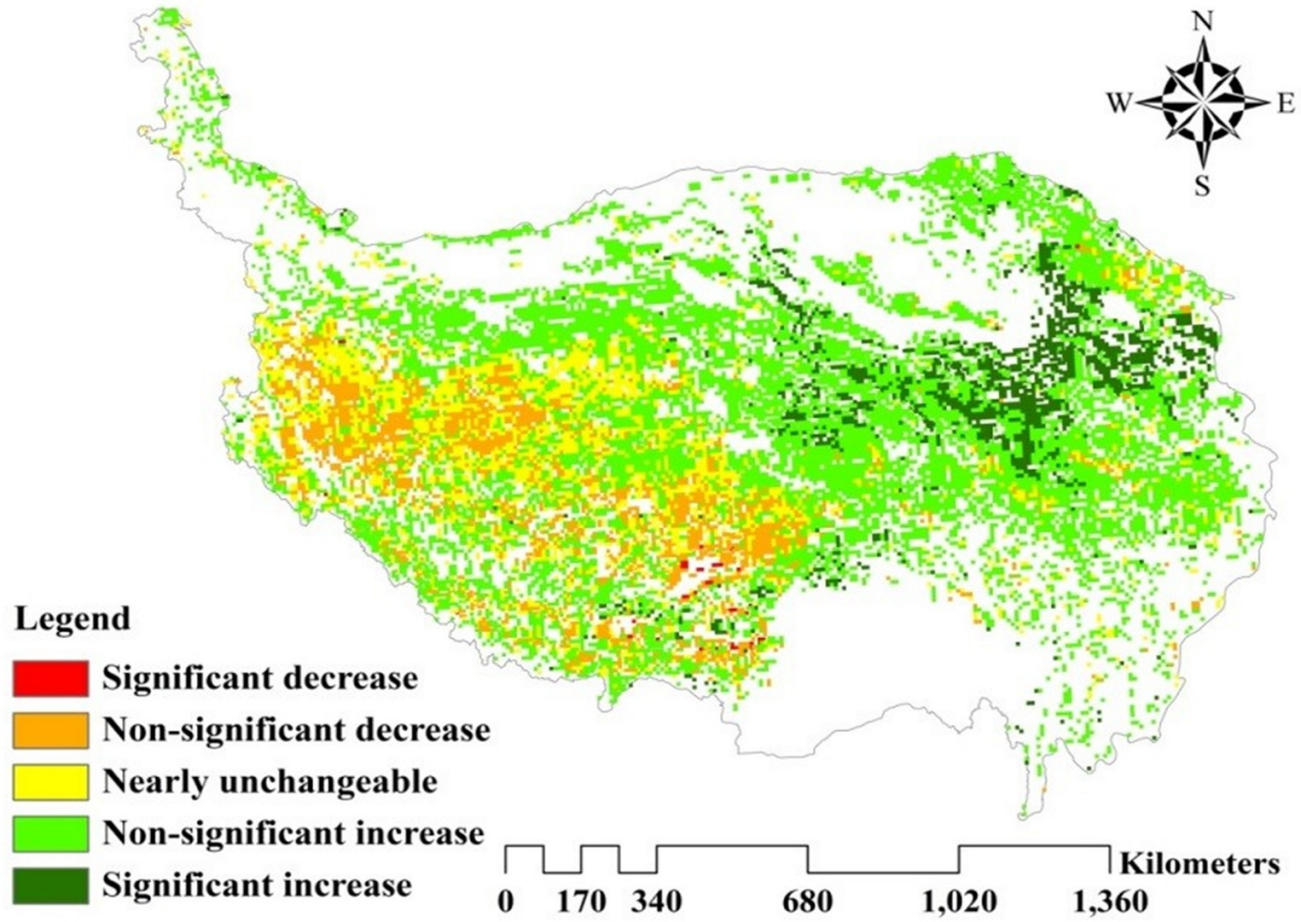
Spatial distribution of SOCD dynamic trends on the QTP during 2000–2015.

To reveal the spatial differentiation in vegetation change, linear regression analysis was used to calculate each grid’s change trend, and all of the grids’ slope values were classified into five levels to evaluate the SOCD change qualitatively (Fig 9, Table 1). More than 60% of the SOCD values in the study area experienced an increasing trend distributed largely in the southeastern area of the Qinghai province. This may be attributable to the combination of the reduced grazing pressure and increased temperatures [43]. Only 14.85% of the grassland in the study area experienced a decreasing trend in SOCD values, most of which was in Tibet, where grassland degradation has occurred in recent years [44]. Of the two grassland types, approximately 83% of the meadow grassland experienced an increasing trend in SOCD; only 7.2% of the meadow SOCD exhibited a downward trend with mean slope values of 0.047 (Table 1). In contrast, the SOCD increased only in approximately 53% of the steppe grassland and over 21.7% of the steppe SOCD experienced a downward trend with an average slope value of 0.021.

**Table 1:** The percentage of meadow and steppe in different categories of change in SOCD during 2000–2015.

Range	Change trend levels	Area percentage (%)		
		Steppe	Meadow	Total
**Slope<-0.1**	Significant decrease	0.44%	0.06%	0.26%
**-0.1≤Slope<-0.01**	Non-significant decrease	21.32%	7.14%	14.59%
**-0.01≤Slope<0.01**	Nearly unchangeable	25.49%	9.73%	18.01%
**0.01≤Slope<0.1**	Non-significant increase	45.62%	71.90%	58.09%
**Slope≥0.1**	Significant increase	7.13%	11.17%	9.05%

### Relation between SOCD and influential environmental factors

**Fig 10:**
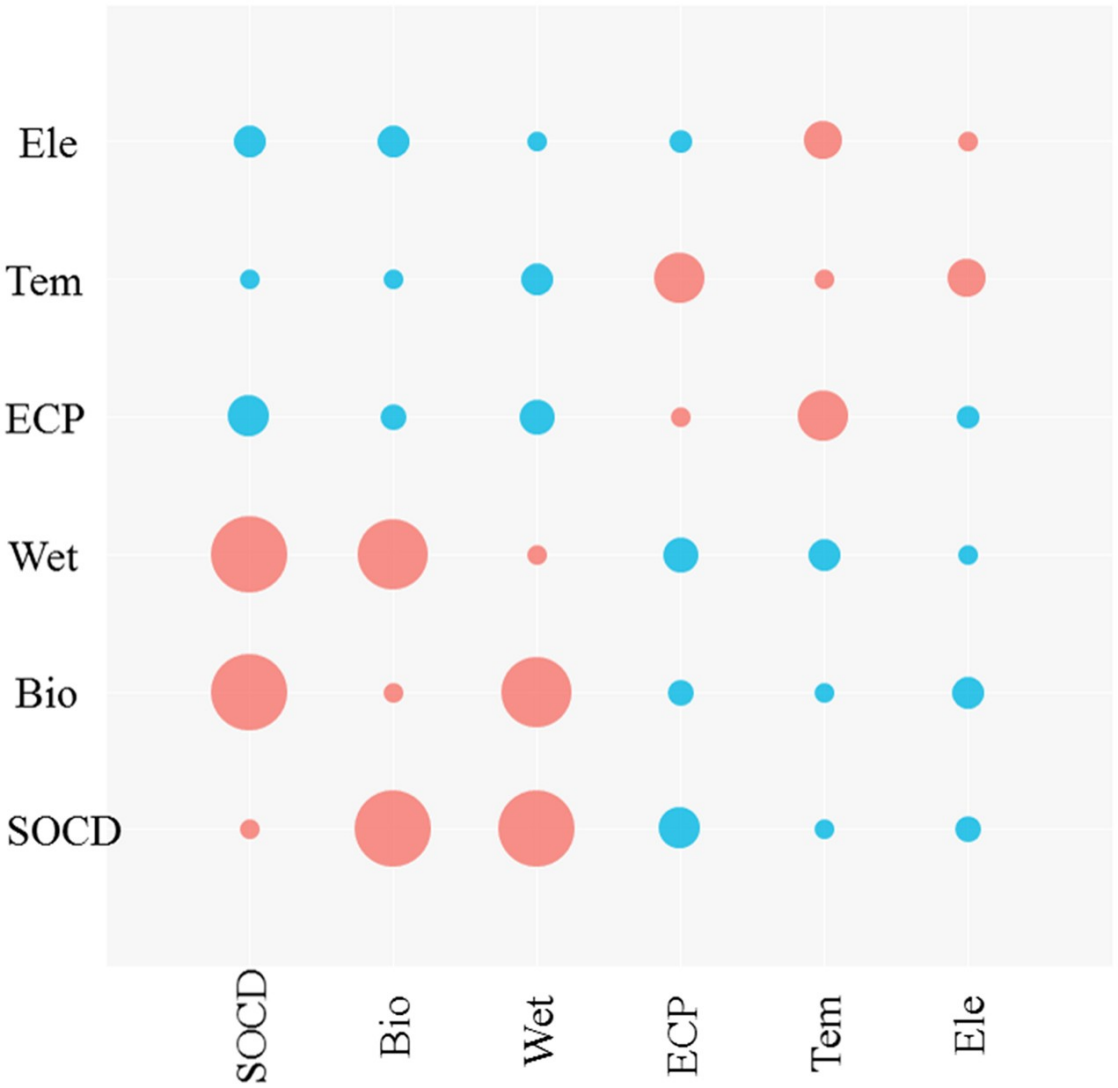
Correlation matrix of SOCD and influential factors for grasslands on the QTP.

Correlation analysis was used to examine the relation between the SOCD values and influential environmental factors (Fig 10). The correlation coefficients between the SOCD and these factors indicated that there were significant positive correlations between SOCD and aboveground biomass, soil moisture, and negative correlations between SOCD and soil conductivity, temperature, and elevation in our study. Correlations between the different factors were also evaluated, and the results indicated that both soil moisture and soil temperature were correlated significantly with soil conductivity, and the latter was correlated negatively with soil moisture and positively with soil temperature. An increase in soil temperature or decrease in soil moisture promoted an increase in soil conductivity, and a significantly negative correlation was found between soil temperature and elevation.

## Discussion

### Comparison of SOCD characteristics between different estimation models

Based on the BD and SOC data, SOCD in this study was calculated between the meadow and steppe grasslands on the QTP using four different models. The results showed that Model3 had a lower SOCD value than did Models1, 2 and 4, which had similar values. The soil profile depth setting may be an important factor that influenced the differences of SOCD estimates in each model. Considering previous studies, Model3 has been generally used to estimate 3-m-deep SOC, while the other three models has been applied to 1-m-deep topsoil to estimate SOC on the QTP [14, 36]. Moreover, SOC was largely concentrated within 60 cm in the topsoil, and SOC content and soil profile depth differed significantly, which led to a lower SOCD value in Model3. This also implied that Models1, 2 and 4 were more feasible to estimate regional SOCD and its change than Model 3 in 30 cm of topsoil on the QTP’s grassland. In addition, the results showed that mean SOCD decreased with increasing soil depth in 30 cm of topsoil, consistent with previous studies [13, 14]. This may be attributable to vegetation root distribution restriction, in that, through root exudates and mortality, roots affected SOC source and distribution trends [15]. Moreover, the photosynthetic allocation to aboveground and underground biomass that fell to the soil surface via branch litter influenced the relative amount of C [15, 45], while the underground biomass of grassland ecosystem decreased rapidly with an increase in soil depth [13].

Further, SOC stocks between meadow and steppe grasslands were estimated using the BD, SOM, and MODIS-NDVI methods, respectively. The similarities in the different grassland types were shown in the similar values in BD 1, 2, 4, and the MODIS-NDVI methods, where the lowest value was in BD03 and the highest in SOM. However, for different grassland types, the different methods differed significantly, with a larger value in the meadow than steppe grassland. The results of this experiment were similar to those of previous studies [13, 46, 47]. Ding et al. (2017) concluded that the alpine meadow’ mean SOCD was higher than that of the alpine steppe in 2000 (2.71 and 6.96 kg cm^-2^, respectively) and 2010 (2.95 and 7.37 kg cm^-2^, respectively) [47]. The soil carbon content of different grassland types on the QTP had obvious zonal differences that were consistent with the distribution of soil and grassland types [46]. The reason for this may be that higher precipitation and lower temperatures influence meadow grassland to a greater extent than steppe grassland, and the decomposition process of SOM is restricted, which results in higher accumulation of SOC. Further, vegetation productivity also may be an important factor that affects SOCD [13, 48]. Generally, the underground biomass of vegetation in high-altitude cold regions was significantly higher than the above-ground biomass. A large number of dead roots and plant residues falls to the soil in the form of SOM that may be converted into large amounts of humus accumulating in cold and humid conditions, resulting in higher SOCD in the meadow grassland [49].

However, compared with steppe grasslands, the MODIS-NDVI for meadow grassland had a lower value of SOCD stocks than did the BD 1, 2 and 4 methods. This may be attributable to higher precipitation and lower temperatures such that meadow grassland has higher vegetation coverage and biomass than does steppe grassland. In the high vegetation-covered area, when the vegetation was becoming denser, NDVI did not increase with the change in biomass and reached light adsorption saturation status with a threshold of 0.8, resulting in a low estimated value in the meadow grassland [27]. However, in the vegetation type where NDVI is saturated, EVI can perform well and reflect the growth status of various vegetation types effectively, even in months with high biomass. Therefore, in future studies, MODIS-EVI data should be obtained to solve the adsorption saturation problem of NDVI.

Spatially, SOCD exhibited a similar variation gradient among Models 1, 2, 3, 4, and the SOM models, with a higher value primarily in sporadic areas in the mid-east. The sporadic distribution of individual high carbon density soils, such as peat soil, gray brown soil and marsh soil, were the main cause of the sporadic distribution of individual high SOCD [14]. However, compared to five other models, MODIS-NDVI demonstrated significant differences with an obvious varying trend that increased from the northwest to southeast. Therefore, based on the value estimated and spatial distribution, the MODIS-NDVI model may be a more suitable method to estimate the SOCD values on the QTP.

### Spatial and temporal trends in SOCD changes on the QTP

With respect to the MODIS-NDVI method, SOCD on the QTP was analyzed both on the spatial and temporal scales. Spatially, SOCD showed significant heterogeneity that increased from the northwest to southeast. The mean distribution of SOCD on the QTP’s grasslands conformed generally to the fact that precipitation on the QTP decreases gradually with the increasing elevation from the southeast to the northwest [14, 50]. This alloplasmatic spatial pattern was largely relevant to the distribution of different grassland types due to the differences in climatic factors and soil properties, which was consistent with previous findings [47, 51]. A greater proportion of steppe grasslands was located in the northwest of the QTP with lower SOCD values. Overall, because of lower precipitation in the desert zone, these areas are unsuitable for vegetation growth and have lower vegetation biomass, which results ultimately in less SOC accumulation [37, 52]. Further, soil types in these areas are grey brown desert soil, brown desert soil, wind sand soil, cold soil and desert saline soil, all of which have lower SOCD, even desert saline with the lowest. However, the highest SOCD value was located in the southeast of the QTP, which contains a greater proportion of meadow grasslands. First, because of more precipitation and lower temperatures, vegetation in these areas grows well and produces higher vegetation biomass, which is conducive to the accumulation of SOC [37, 52]. Further, black soil is distributed in these areas, and has the highest SOC content, followed by mountain meadow soil, marl soil, peat soil, brown coniferous forest soil, and bog soil and so forth with higher SOCD.

Temporally, the annual SOCD for different grassland types exhibited increasing trend from 2000 to 2015 in more than 60% of the QTP, which was consistent with previous studies [17, 19, 47]. Ding et al. (2017) found an overall accumulation of SOC regardless of vegetation type across Tibetan permafrost regions [47]. This may be attributable to the increased vegetation productivity that climate change has caused [34, 43]. Over the past several decades, the QTP has experienced warming, higher precipitation, and permafrost thawing, which has induced changes in the ecosystem’s C processes, such as C-gain attributable to stimulated vegetation productivity and C-loss associated with accelerated SOM decomposition [53–55]. Overall, vegetation C inputs’ increase climate caused outweighed soil C losses, which has resulted in a clear increase in SOC stock on the QTP [47]. Through the Mann-Kendall (M-K) test, Z = 4.50>1.96, β>0 indicating that the SOCD showed a significant upward trend. Meanwhile, the meadow grassland showed a faster increase than the steppe grassland. Consistent with our findings, Ding et al. (2017) also concluded that the mean increase rates were 20.6 and 40.3 g cm^-2^ yr^-1^ for the steppe and meadow grassland, respectively [47]. SOC content’s variation and distribution in different types of grassland depends on plant communities and soil moisture on the QTP [13, 46]. Increasing plant productivity attributable to high vegetation coverage and increasing precipitation are the main cause of the rapid increase in SOC in the alpine meadow.

### Environmental factors’ influence on SOCD

In this study, a correlation analysis was performed to identify environmental factors’ effect on SOCD on the QTP, including aboveground biomass, soil moisture and soil conductivity, temperature, and elevation. The results showed that SOCD concentration was associated with the balance between SOM input and loss [1]. Our results showed a positive correlation between SOC and aboveground biomass, a finding consistent with previous studies of alpine ecosystems on the QTP [1, 26]. Increasing plant production may be the principal factor that leads to increased SOC density, which indicates that vegetation biological processes improve SOC accumulation in cold alpine grasslands regions [26, 27]. Further, the positive correlation between SOCD and aboveground biomass demonstrated the effectiveness of the application of the remote sensing dataset to evaluate SOCD [17, 27].

A significant positive correlation between the SOCD and the soil moisture was observed, which was consistent with previous studies performed on the QTP [18, 26, 52]. This result may be attributable to the fact that the soil moisture is associated with organic matter decomposition rate and vegetation biomass [18]. Studies have found that, as soil moisture increased, the aboveground vegetation and root biomass increased, and the decomposition rate of ground litter and fine root in the shallow roots also increased with increased soil water content [56]. Generally, more precipitation can improve plant production and soil decomposition, both of which contribute to the accumulation of SOC in a water limited area [18, 57]. Therefore, soil moisture has an important effect on the surface SOC content, which increased significantly with increased water content.

Increasing temperatures resulted in accelerated decomposition of soil material and thus decreased SOC [58]. Therefore, temperature is also an important factor that influences SOCD [59]. In our study, SOCD was correlated negatively with temperature. This result is consistent with the global changes and some studies at regional scales between 52°N and 40°S [18, 60]. In our study, a 1°C increase in temperature resulted in a 0.36% decrease of SOCD, which is similar to the 3.3% decrease of SOCD at the global level. Temperature is a limiting factor for soil materials in the alpine grasslands on the QTP and higher temperatures may accelerate soil decomposition, and thus, cause SOCD to decline [15]. This implies that soil processes constrain SOCD accumulation in cold regions, which was consistent with the findings from most of the regions. However, it contrasted with those in many studies on the QTP [9, 18, 27], which concluded that the increasing C inputs may exceed C losses induced by temperature in the accelerated decomposition process with increasing temperature, and thus SOCD increased [18, 58]. Therefore, a consensus about the relation between SOCD and temperature at a regional scale is still lacking and should be explored deeply in future studies.

Elevation is another complex factor of SOCD that controls the temperature and precipitation changes along elevation gradients. Generally, temperature tends to become lower with increasing altitude, which would limit SOM’s decomposition rate and accelerated SOCD accumulation. Many studies found that SOCD was correlated positively with elevation [24, 61, 62]. However, our study found the opposite result, with a negative correlation between SOCD and elevation, which was consistent with the results in the northern the agro-pastoral ecotone [21], the dammed field in the Loess Plateau [63], and the alpine shrubland in the Three Rivers Source Region [18]. This may be attributable to the narrow range of elevation and the distribution of vegetation along elevation gradients [21]. In our study, the elevation was high and ranged to more than 3000 m, and different vegetation distributions may be the primary reason for the negative correlation between SOCD and elevation, in that forest and grassland were located primarily in low-elevation areas, while large areas of sandy land were distributed in high-elevation areas [21]. Further, the SOCD was correlated negatively with soil conductivity, a result was inconsistent with previous studies of the desert grasslands [64].

### Uncertainties and limitations

In this study, BD, SOM and remote sensing datasets were used to estimate the spatio-temporal SOCD change in the steppe and meadow on the QTP from 2000 to 2015. In comparison with the BD and SOM models, the MODIS-NDVI method showed significant spatial differentiation with valid values, which may reduce the uncertainties that soil’s spatial heterogeneity caused. However, SOCD estimation in this region remains uncertain because of the influence of data sources, sample size, and other factors. Firstly, because of the special weather and topographical conditions that make certain areas inaccessible, most soil sampling sites were located in the mid-altitude areas of the QTP, which may have resulted in uneven or insufficient sampling points. There also potential uncertainties about the regression relations that form spatial patterns of SOC density [7, 17]. Secondly, the MODIS-NDVI method could account for larger proportional differences in SOCD, but some uncertainties resulted from the remaining residuals that may be introduced into SOCD regional estimation [7, 17]. Thirdly, limited by time, human resources and funding, only 9 soil profiles were collected at each sampling point. Further, when the Kriging space interpolation was conducted, the different soil types’ spatial characteristics and areas were not considered fully, which may cause a certain degree of deviation in SOCD estimates on the QTP. Finally, in our study, we considered only aboveground biomass, soil moisture and conductivity, temperature, and elevation’ influence on SOCD, while many other environmental factors were not considered, such as land use and soil types, soil texture, vegetation cover, slope, and aspect, among others [12, 65]. For example, land use changes are a very important factor that influences regional carbon dynamics, and was found to account for 17% of the carbon increase at the national level [66, 67]; Wang et al., concluded that SOCD was correlated significantly and positively with silt and clay contents, while it was correlated significantly and negatively with sandy soil [68]; Chen et al., indicated that SOC stocks on shady slopes increased with MAT at high elevations, but decreased with MAT in low-lying areas [58]. Therefore, more ground-based soil data and environmental factors should be investigated and considered to improve the accuracy of SOC storage estimation in future work.

## Conclusions

In this study, based on BD, SOM and remote sensing datasets, we estimated the spatio-temporal changes in SOCD between steppe and meadow grasslands on the QTP from 2000 to 2015. The BD dataset of different models showed similar variations in SOC estimation when the values ranged from 1.1 to 1.4. Compared with the great differences between BD 3 and SOM methods, the MODIS-NDVI, and BD 1, 2 and 4 methods yielded close and valid values of SOC storage on the QTP’s grassland. Principally, SOC storage was greater in the meadow grassland than in the steppe grassland. Spatially, compared with the five other methods, the MODIS-NDVI method showed significant spatial heterogeneity that increased from northwest to southeast. Therefore, considering the values estimated and the spatial distribution trend comprehensively, the MODIS-NDVI method may be a more accurate and appropriate model to estimate SOCD. Temporally, based on the MODIS-NDVI model, the steppe or meadow’ annual SOCD all showed increasing trends during the study period, and the slope value was calculated to reveal SOCD’s spatial change differences. More than 60% of the areas distributed in the southeastern regions of the Qinghai province showed an increase in the two grassland types, and the meadow experienced a more significant increase in SOCD than did the steppe grassland. Further, SOCD showed significant positive correlations with soil moisture and aboveground biomass, indicating that these two factors were the main factor that affected affecting soil carbon stock on the QTP. The SOCD had a clear negative correlation with soil conductivity, while it was correlated weakly with elevation and temperature. In future research, additional environmental factors should be included to elucidate the mechanisms of variations in SOCD’s spatial and dynamic distribution on the QTP.
